# Draft Genome Sequence of Psychrotrophic *Acinetobacter* sp. Strain MN12 (MTCC 10786), Which Produces a Low-Temperature-Active and Alkaline-Stable Peptidase

**DOI:** 10.1128/genomeA.01167-14

**Published:** 2014-11-20

**Authors:** Mohit Kumar Swarnkar, Richa Salwan, Ramesh Chand Kasana, Anil Kumar Singh

**Affiliations:** a Division of Biotechnology, CSIR- Institute of Himalayan Bioresource Technology, Palampur, India; b Hill Area Tea Science Division, CSIR, Institute of Himalayan Bioresource Technology, Palampur, Himachal Pradesh, India; c Academy of Scientific and Innovative Research, New Delhi, India

## Abstract

We report here the draft genome sequence of *Acinetobacter* sp. strain MN12 (MTCC 10786), which is a psychrotrophic bacterium that produces an extracellular low-temperature-active and alkaline-stable peptidase. The draft genome assembly of *Acinetobacter* sp. MN12 has a size of 4.31 Mbp, with a G+C content of 40.75%.

## GENOME ANNOUNCEMENT

Previously, we isolated *Acinetobacter* sp. strain MN12 from soil from the Mane area (32°1′42″N 78°14′22″E) of Lahaul and Spiti, India (1). The protease produced by *Acinetobacter* sp. MN12 is active at low temperatures, alkaline stable, and effectively removes blood stains at a low temperature (2). The culture was deposited at the Microbial Type Culture Collection and Gene Bank, Chandigarh, India (accession no. MTCC 10786). The genome of *Acinetobacter* sp. MN12 was sequenced, given its ability to grow and produce low-temperature-active and alkaline-stable proteases of commercial importance.

Whole-genome shotgun sequencing of *Acinetobacter* sp. MN12 was completed using the Illumina Genome Analyzer IIx in 76-bp paired-read format. We obtained 21,418,969 raw reads, with 3,255,683,288 bp of raw sequence. The paired reads were quality filtered using the NGS QC toolkit version 2.3 (3). The cutoff for quality score is >20 Q30 and should have high-quality bases of >70% of read length. A total of 16,615,129 (77.57%) of the filtered reads were obtained without adaptor/primer contamination, and these were used for assembly. *De novo* assembly of the genome data was done using ABySS version 1.3.7 (4). In this data set, the k parameter (from 43 to 73) was optimized for best assembly. We found that at k = 69mers, 72.5% (12,053,547) of the reads were found paired, and 24.1% (3,999,305) of the reads were singletons. We used SSPACE version 3.0 (5) to extend and merge the resulting scaffolds based on read-pair information and short overlaps to reduce the number of contigs. GapFiller version 1.1 (6) was used to close the gaps between the short contigs that are contained within the large contigs by replacing unknown nucleotides (Ns) with true nucleotides based on read-pair information and short overlaps. After filling the gaps, the reads were assembled as 107 contigs summing 4,314,475 bp (*N*_50_ size, 107,558; longest size, 249,707; G+C content, 40.75%). Annotation was conducted with the RAST server using the Glimmer 3 option and predicted 4,074 protein-coding genes, including 83 RNA genes and 403 predicted SEED subsystem features (7). A total of 1,594 (40%) features were covered by seed subsystems, out of which 1,530 were nonhypothetical proteins. The annotation of the MN12 genome using Prodigal (8) predicted 4,017 coding sequences (1,407 >1 kb; mean size, 935) covering 87% of the assembled genome. Further, tRNAscan-SE (9) and RNAmmer (10) predicted 78 noncoding RNAs (73 tRNAs, 2 pseudo-tRNAs, and 3 rRNAs consisting of 3 copies each of 5S rRNA, 16S rRNA, and 23S rRNA genes).

A comparison of the MN12 genome with existing genome sequences performed using the RAST server revealed that *Acinetobacter junii* SH205 (score, 501) and *Acinetobacter haemolyticus* ATCC 19194 (score, 477) are its closest neighbors. In the MN12 genome, 327 genes were assigned for amino acids and derivatives, 215 for carbohydrates, and 207 for protein metabolism. The genome contains 66 genes predicted for proteases and peptidases. The comparative genome analysis of *Acinetobacter* sp. MN12, a protease-producing psychrotroph, with other *Acinetobacter* species will give insight into the evolutionary relationship and knowledge about the basic genetics of this important genus.

### Nucleotide sequence accession numbers.

This whole-genome shotgun project has been deposited at DDBJ/EMBL/GenBank under the accession no. JROB00000000. The version described in this paper is version JROB01000000.
